# Enhancement of Vibronic and Ground-State Vibrational Coherences in 2D Spectra of Photosynthetic Complexes

**DOI:** 10.1038/srep02029

**Published:** 2013-06-19

**Authors:** Aurélia Chenu, Niklas Christensson, Harald F. Kauffmann, Tomáš Mančal

**Affiliations:** 1 Faculty of Mathematics and Physics, Charles University in Prague, Ke Karlovu 5, 121 16 Prague 2, Czech Republic and; 2 Faculty of Physics, University of Vienna, Strudlhofgasse 4, 1090 Vienna, Austria

## Abstract

A vibronic-exciton model is applied to investigate the recently proposed mechanism of enhancement of coherent oscillations due to mixing of electronic and nuclear degrees of freedom. We study a dimer system to elucidate the role of resonance coupling, site energies, vibrational frequency and energy disorder in the enhancement of vibronic-exciton and ground-state vibrational coherences, and to identify regimes where this enhancement is significant. For a heterodimer representing two coupled bachteriochloropylls of the FMO complex, long-lived vibronic coherences are found to be generated only when the frequency of the mode is in the vicinity of the electronic energy difference. Although the vibronic-exciton coherences exhibit a larger initial amplitude compared to the ground-state vibrational coherences, we conclude that, due to the dephasing of the former, both type of coherences have a similar magnitude at longer population time.

The observation of long-lived oscillations in the two-dimensional spectra of Fenna-Mattews-Olson (FMO) pigment-protein complex^1^ has renewed the general interest in quantum effects in light-harvesting and other biological systems. Although similar observations were reported previously^2^,^3^, the interpretation of the oscillations as electronic coherence and the suggestion that such dynamical coherence may play a crucial role in achieving the high efficiency of electronic energy transfer in photosynthesis^1^ have ushered a wave of research on the role of coherence in excitation energy transfer processes.

Numerous theoretical articles have been devoted to understanding and explaining the long-lived oscillations in FMO. They have shown that coherent oscillations last for several hundreds of femtoseconds, but under standard assumptions about the properties of the protein environment, none of these works have been able to account for the over picosecond long dephasing times of the oscillations observed experimentally^4^,^5^,^6^. The only proposed mechanism which predicts electronic coherence with life times >1 ps is the correlation between environmental fluctuations on different pigments^4^,^5^,^7^,^8^,^9^,^10^,^11^. Experimental confirmation of correlated distributions of pigment energies has been claimed for FMO^12^, but neither dynamic nor static correlation of the pigment energies have been found in molecular dynamics (MD) simulations of the FMO protein environment^13^,^14^. Furthermore, dynamic correlation, if present, would lead to an effective decrease of the system bath coupling strength, and to a corresponding slowdown of the energy transfer rates. Such a decrease of the system-bath coupling strength is at odds with works which find an optimal strength of the system bath interaction for transport function within the parameter range used by standard theories^14^,^15^. Also, the energy transfer rates obtained by standard theory are in a good agreement with experimental data^16^, and the standard “funnel picture” of energy transfer^17^ seems therefore to be well supported.

The assumption that the experimentally observed coherences would be relevant to the biological function of the antenna complexes has also been criticized. Such a proposal requires the excitation with coherent superpositions of states created by lasers in the laboratory to be, in some way, equivalent to the excitations under *in vivo* conditions^18^, i.e. by direct sunlight or via transfer from another antenna system. However, serious objections to this view have been raised in the literature, arguing that direct excitation by light from the sun (thermal light) does not lead to such coherent excitation^19^,^20^,^21^,^22^,^23^. A recent experiment showed that even under coherent excitation of the chlorosome antenna, i.e. with femtosecond laser pulses, no coherent oscillations could be observed^24^. This implies that any coherence induced by the excitation would decay quickly, and that the energy is transferred to the FMO complex in an incoherent fashion independently of the excitation conditions.

The above discussion suggests that the mechanism explaining the oscillations does not need to have a strong impact on the energy transfer dynamics or be relevant to the energy transfer efficiency. The problem should therefore be studied as a problem of oscillations in experimental signals rather than a problem of oscillations of electronic excited-state populations as it is often presented. In other words, the origin of the oscillating experimental signal has to be understood before the signal can be translated into claims about the energy transfer dynamics. Recently, Christensson *et al.*^25^ proposed that the excitonic interaction between electronic and vibrational states in FMO serves to create vibronic states (excitonically mixed electronic and vibrational states). Such states have a considerable vibrational character, and, at the same time, have an enhanced transition dipole moments due to intensity borrowing from the strong electronic transitions^26^. It was also shown that coherent excitation of the vibronic states produces oscillations in the non-linear signal that exhibit picosecond dephasing times. Moreover, the concept of vibronic excitons provides a plausible explanation for the observation of correlated distribution of site energies^12^. The vibronic-exciton states involved in the long-lived coherences are to a large extent composed of different vibrational states on the same pigment. This automatically leads to a correlation in the fluctuations of the involved transitions even for a random distribution of pigment energies.

The influence of vibrational degrees of freedom (DOF) on dynamics of excitonic systems has currently become an intensely studied topic. Recent research suggests the coupling between excitons and vibrations to have a key role in the dynamics of coherent light harvesting in cryptophyte algae^27^. Also, the vibronic-exciton model has been shown to provide, compared to the purely excitonic model, more realistic relaxation rates, as verified against experimental measurements in two cyanobacterial light-harvesting proteins^28^. In addition, recent simulations suggest the existence of mode-driven coherences, where an initial excitation of an underdamped bath mode is resonantly transfered to and sustains long-lived (ps) coherent oscillations between electronic eigenstates^29^.

In a recent work, Tiwari *et al.*^30^ used a model similar to the vibronic-exciton model used here to show that the mixing of electronic and vibrational DOF leads to an enhancement of the excitation of vibrational coherences in the electronic ground state as well, and it was also argued that this effect can explain the long-lived oscillations in FMO. In order to distinguish between the coherences observed in the excited-state manifold and the ones originating in the ground-state manifold, we will assign the term *vibrational* coherence strictly to the latter ground-state contribution. There, the states are of pure vibrational origin although the ability to excite them is provided by the mixing of vibrational and electronic states in the excited-state manifold. When referring to the excited-state coherences with a strong vibrational involvement, we will use the terms *vibronic*, or more specifically *vibronic-exciton* coherences.

In this paper, we turn to a model system (an FMO-inspired molecular dimer) in order to systematically investigate how the mixing of vibrational and electronic DOF leads to long-lived oscillatory signal in non-linear optical spectroscopy. The paper is organized as follows. In the next section, we explain the mechanism of coherence amplitude enhancement on a toy model. Then, we introduce the model dimer system with the associated notation, and we define a measure of the vibrational character of a coherence. A section on 2D non-linear spectroscopy introduces the third-order non-linear signal resolved in the coherent 2D Fourier transformed spectroscopy and its relation to the dynamics of molecular systems, in particular to its coherent dynamics. After determining the basic properties of the enhancement for vibronic (excitonically mixed electronic and vibrational) and the ground-state vibrational coherences on an FMO-inspired heterodimer, we discuss the results in context of FMO and the low frequency vibrational spectrum of BChl-a.

## Results

### Enhancement of coherence amplitude by transition dipole moment borrowing: a toy model

**Figure 1:**
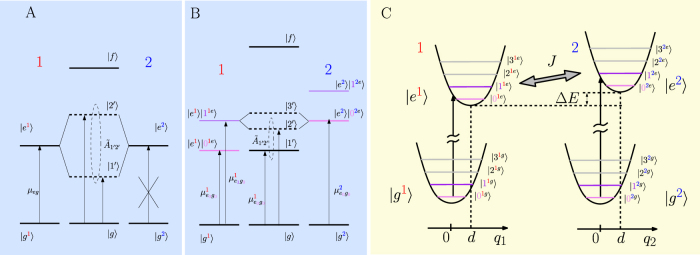
Illustration of the effect of transition dipole moment borrowing and excitonic mixing of excited states in a dimer without considering vibrational states (A) and considering a simplified model of vibrational presence (B). In the case (B), a heterodimer is considered with an approximate resonance between the one-phonon state of monomer 1 and the zero-phonon state of monomer 2. Panel (C): Dimer model with a single vibrational mode per monomer. The monomers 1 and 2 can be in their respective ground states |*g*〉 or excited states |*e*〉. In each electronic state, the monomers can occupy any of the vibrational levels corresponding to their respective vibrational modes (*q*_1_ or *q*_2_).

To illustrate the mechanism of enhancement of the coherence amplitude that is suggested in Ref. 25 and studied in detail in this paper, let us first consider qualitatively a rather trivial example. Let us imagine a system of two molecules, one of which has a forbidden transition to the excited state. In the absence of excitonic coupling between the excited states of the two molecules, it is not possible to excite a linear combination of the collective excited (eigen)states of the dimer (i.e. the coherence observed by non-linear spectroscopy), because one of the transitions is forbidden. If we now switch on the interaction between the monomers, it becomes possible to excite the coherence between the new eigenstates of the system (see Fig. 1A for illustration). We assume the transition dipole moment in a form 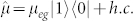, where |1〉 = |*e*^(1)^〉|*g*^(2)^〉 is the allowed collective excited state of the dimer with the monomer 1 in its excited state |*e*〉 and monomer 2 in its ground state |*g*〉 and |0〉 = |*g*^(1)^〉|*g*^(2)^〉 is the collective ground state (here, the upper index (*n*) refers to the *n*-th monomer). Expressed in the new eigenstate basis of vectors |*α*〉, the transition dipole moment reads 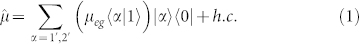
The new transition dipole moment to the excited eigenstates |*α*〉 reads 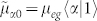. As will become clear in the following sections, the amplitude of the contribution of a coherence between two states |*α*〉 and |*β*〉 to the 2D spectrum is proportional to the factor 
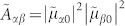
. In a dimer, we have only eigenstates |1′〉 and |2′〉, i.e. only one coherence, and we obtain 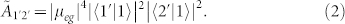
Because |〈1′|1〉|^2^ + |〈2′|1〉|^2^ = 1, the maximum value of 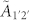 is equal to 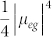 when the mixing is maximum, i.e. 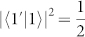.

In a dimer, the coefficient 〈1′|1〉 is given by a sine or cosine of the mixing angle 
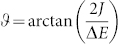
, where *J* is the resonance coupling and Δ*E* is the difference between energies of the interacting states^31^. When *J* is small, the mixing can only be substantial at the resonance, i.e. when Δ*E* → 0. The observation of coherence in 2D spectroscopy depends crucially on the ability to excite the two involved excited states simultaneously. In the above case, this is enabled by the resonance between excited states.

In Fig. 1B, we present another interesting case. We extend the previous toy model with vibrational states at each monomer, and assume both monomers to have allowed transitions to the excited states. Each state on the molecule (*n*) is characterized by its electronic manifold (*e* or *g*) and its vibrational level ν, e.g. 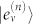. In Fig. 1B, only the first vibrationally excited state in the electronically excited state is represented (ν ≤ 1). The one-phonon state of monomer 1 interacts weakly with the zero-phonon electronically excited state of monomer 2. Because the coupling is small, the interaction between the two zero-phonon states of the interacting monomers can be neglected, and the borrowing effect occurs only for the collective states |2′〉 and |3′〉 of Fig. 1B. If, in addition, we assume the transition dipole moments to the one-phonon state to be small (i.e. the vibrational mode has a low Huang-Rhys (HR) factor), we obtain the situation similar to Fig. 1A. At resonance (i.e. at maximum mixing 
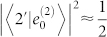
), the coherence amplitude 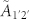 in 2D spectrum reads 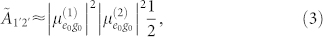
where 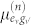 is the transition dipole moment between the electronic excited state in the vibrational level ν and electronic ground state in the vibrational level ν′ of molecule *n*. This is to be compared with the amplitude of the purely vibrational coherence on a noninteracting monomer which is 
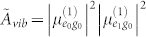
 and which is small due the small HR factor. The excitonic interaction thus enhances a coherence which has partially both the vibrational and the electronic characters.

The situation studied later in this paper is similar to the model illustrated in Fig. 1B. We will study coherences involving the zero- and one-phonon states of the same vibrational mode on a given monomer. As demonstrated, the transition from the electronic and vibrational ground state to the one-phonon state can be enhanced by excitonic coupling with the allowed states of other monomers. According to Ref. 25, this is the case for FMO, where the enhanced coherences were found to be of a prevailingly vibrational character, thus exhibiting significantly prolonged life time over the purely electronic coherences.

The excited-state mixing between the vibrational states of one monomer and the electronic (zero-phonon) state of the other monomers can, however, enhance also the ground-state bleaching signal^30^, which was not included in our toy model. The life-time of the coherence has not been discussed within the toy model either, because it is not relevant to the illustration of the enhancement mechanism. However, it will be of crucial importance throughout the rest of the paper.

### Dimer hamiltonian: full formulation

In this paper, we concentrate on the relatively strongly coupled dimer of bachteriochloropylls-a (BChls-a) 3 and 4 of the FMO complex^32^, which we will later refer to as monomer 1 and monomer 2, respectively. Needless to say, the interaction of the other BChls in the complex with this dimer is decisive for its function as a molecular wire, and it influences the properties of the oscillations observed in 2D spectra as well. In Ref. 25, it was demonstrated that the effect of long-lived coherence could plausibly originate from the interplay between the vibrational modes local to BChl 3 and the resonance interaction between BChls 3 and 4. By reducing FMO to this dimer, we expect to be able to isolate the main quantitative contributions to the observed effect, and to simplify the treatment such that the main conditions for the effect to take place can be identified. The extension of the results to the entire FMO complex is addressed in the discussion.

The studied molecular system will be represented by a dimer Hamiltonian, explicitly treating a single intramolecular vibration (with frequency *ω*_0_) on each of the monomers. Unlike in Ref. 25, we will first consider the formulation with an arbitrary number of vibrational-excited levels, which we refer to as a full formulation (full with respect to the number of vibrational levels). For practical calculations, we will then reduce the number of vibrational levels to a single excited level on each monomer. This is justified by the low Huang-Rhys factors of the vibrations in chlorophylls^33^. The dimer Hamiltonian in the electronic site basis is defined as 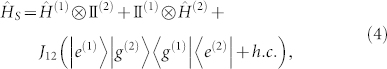
where 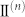 represents the identity operator on the Hilbert space of monomer *n*, and 
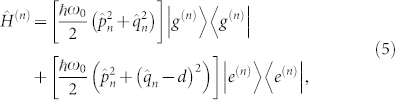
is the Hamiltonian of monomer *n*. Here 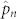 and 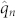 are the dimensionless momenta and coordinate operators of the vibrational mode on site *n*, respectively, *d* is the coordinate displacement, assumed identical for all monomers, and *J_nm_* denotes the inter-site coupling. The corresponding level model including the vibrational states and the associated notation is presented in Fig. 1C.

This system interacts with a bath of protein DOF which is modeled as an infinite number of harmonic oscillators characterized by some continuous spectral density, e.g. 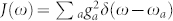. The Hamiltonian representing the coupling between the bath modes characterized by the Hamiltonian 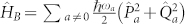, and the dimer reads as 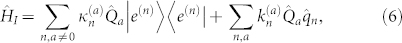
where the mode *a* = 0 has been excluded since it is explicitly treated in the molecular Hamiltonian. The first term in Eq. (6) corresponds to the linear interaction between the bath and the electronic DOF, and it can be expressed through the spectral density as 
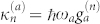
32. The second term in Eq. (6) describes a bi-linear interaction between the intramolecular mode and the bath. On the basis of experimental measurements showing lifetimes on the order of a few ps for the low frequency vibrational coherences in BChls^34^, we will consider this interaction to be weak, and we remove any bath-induced dephasing of the vibrational modes by setting 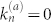 for all *n*, accordingly.

Let us now define the notation used throughout this paper to describe the dimer state. As in the standard electronic molecular exciton theory, the excited states of the dimer are formed from the excited states of the monomers. We denote the electronic excited states by a diad (*nm*) where *n* (*m*) stands for the state (*g* or *e*) of monomer 1 (2) in correspondence with Fig. 1C. The Hilbert space in which the molecular Hamiltonian will be represented can be fully described by states |*n*_ν_*m*_ν′_〉, where the first (second) letter describes the first (second) monomer's electronic state, and the index ν (ν′) denotes its respective vibrational quantum level, so that e.g. |*e*_ν_*g*_ν′_〉 = |*e*^(1)^〉|ν^(1*e*)^〉|*g*^(2)^〉|ν*′*^(2*g*)^〉. The eigenstates of the Hamiltonian will be written as linear combinations of the states |*n*_ν_*m*_ν′_〉, considering the singly-excited states only, i.e. 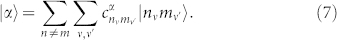
The expansion coefficients obtained by diagonalization of the Hamiltonian 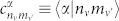 will report on the participation of the particular local states in the eigenstates. The eigenstates will be numbered with increasing energy 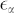, *α* = 1,2,….

### Measure of the vibrational character

To measure the vibrational character of a coherence, we can use the square of the expansion coefficients. We will be interested in the coherence between states |*e*_0_*g*_ν′_〉 and |*e*_1_*g*_ν′_〉. This coherence is a vibrational coherence located on monomer 1, and we ignore the vibrational state of the second monomer. Because, in the low temperature approximation, the ground-state vibrational quanta of the second monomer cannot be excited from the thermal ground state by a single interaction with the exciting pulse in the optical domain, we can set ν′ = 0 without loss of generality. We are interested in identifying which coherences between the eigenstates have the character of this local vibrational coherence, and we want to define accordingly a measure *χ_αβ_* of the *local vibrational character* of the coherence *ρ_αβ_*. For a given coherence to be vibrational on monomer 1, the state |*α*〉 needs to have a character of the zero-phonon state on the monomer 1, i.e. it has to be predominantly composed of the state |*e*_0_*g*_0_〉, and the state |*β*〉 has to have predominantly the character of the state |*e*_1_*g*_0_〉 (or *vice versa*). This composition is measured by the square of the corresponding expansion coefficients 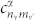 and 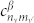, which also define the probability of finding the system in the corresponding local states, should we attempt such a measurement. The character of the coherence corresponds to the conditional probability of finding the state |*e*_0_*g*_0_〉 by measuring on state |*α*〉, and of simultaneously finding the state |*e*_1_*g*_0_〉 by measuring on state |*β*〉, or *vice versa*. This leads us naturally to the definition 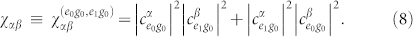
This quantity has its maximum when the eigenstates are each entirely composed of the zero-or one-phonon states of the excited molecule, and thereby correspond to the site basis states (*χ_αβ_* = 1 for |*α*〉 = |*e*_0_*g*_0_〉 and |*β*〉 = |*e*_1_*g*_0_〉, or *vice versa*). When the states |*α*〉 and |*β*〉 are equal mixtures of these local states, so that 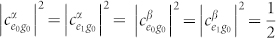, then we have 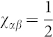. Therefore, coherences *ρ_αβ_* with 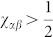 will be considered to have *prevailingly local vibrational character*. We compared the local vibrational character defined by Eq. (8) with the composition of the eigenstate vectors 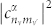, and we verified that, regardless of the variable used to describe it, we reach the same conclusion about the character of a given coherence. Note that the character *χ_αβ_* is a time-independent quantity. It refers to eigenstates of the Hamiltonian and their representation in the basis of states local to the chromophores. The coherences have life times dependent on the interaction of the system with the bath, but their local vibrational character *χ_αβ_* only depends on the system Hamiltonian and does not evolve in time.

### 2D non-linear spectroscopy and Liouville pathways

Non-linear spectroscopy has been widely used to study the dynamics of excitonic energy transfer in light-harvesting systems, in particular because it is sensitive to the time dependent redistribution of the populations among excited states. Details about this class of measurement techniques are outside the scope of this work, and they can be found elsewhere in literature (see e.g. Ref. 35,36,37). Two-dimensional (2D) coherent spectroscopy, which is one of the recent additions to this class of techniques, has enabled us to directly observe the coherent components of the excited-state dynamics^38^,^39^. In 2D coherent electronic spectroscopy, the time dependent signal (the detection time is usually denoted as *t*_3_ here) is generated from the interaction of the sample with three consecutive ultrafast laser pulses with wave vectors ***k***_1_, ***k***_2_ and ***k***_3_ separated by time intervals *t*_1_ and *t*_2_^38^,^40^. The signal is heterodyne detected, i.e. the generated field rather than its intensity is measured, and it is spectrally resolved in frequency *ω*_3_ (*t*_3_ and *ω*_3_ are correspondingly related by Fourier transform). The measured field is then numerically Fourier transformed in time *t*_1_ (*t*_1_ → *ω*_1_) resulting in a signal dependent on two frequencies *ω*_3_ and *ω*_1_ and one time delay *t*_2_. 2D spectra are represented as frequency-frequency correlation plots at various time delays *t*_2_. The 2D signal is related to the third-order polarization of the molecular system generated by the exciting laser pulses, and its theoretical description is most conveniently based on the non-linear response function formalism in the third-order of perturbation theory (see e.g. Ref. 41). In this section, we will briefly present the various signal components that can be distinguished in a 2D spectrum, and we will give some of their characteristics, such as positions in the 2D spectrum, oscillation frequency, initial- and time-dependent amplitudes.

**Figure 2:**
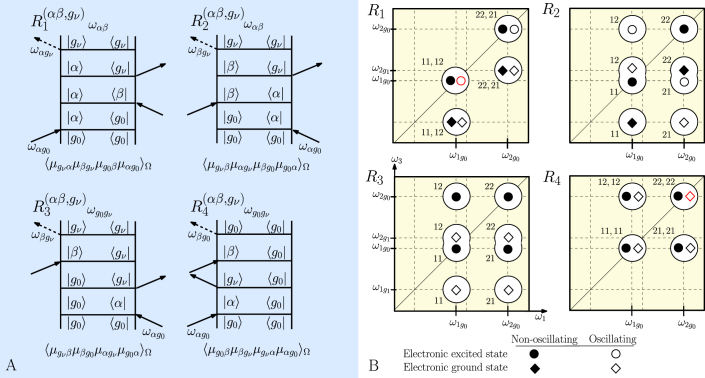
Liouville pathways and their respective contribution to the 2D spectrum. (A) Double-sided Feynman diagram describing the four Liouville pathways along with their dipole pre-factor, the frequency of the involved coherence and the frequency of the exciting and probing pulses. (B) Position of the signal in the 2D spectrum and its characteristics originating from the different pathways. Solid symbol denote non-oscillating contributions in *t*_2_, open symbols denote oscillatory contributions with frequencies *ω_αβ_* (circle), 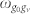 (diamond). Signals denoted by circle symbols refer to the electronic excited-state manifold and do not involve any (purely) vibrational level whereas signals on the electronic ground-state manifold, denoted by diamonds, do (for ν ≥ 1). The diads in (B) represent the eigenstates |*α*〉, |*β*〉 involved in each signal contribution. The red contours highlight the signals for which we present detailed results below.

Depending upon the time ordering of the light-matter interactions in the third-order response functions, four different types of so-called Liouville pathways (denoted *R_i_*, *i* = 1,…,4) can be distinguished in the generated signal. They are represented in Fig. 2A by double-sided Feynman diagrams^41^. Pathways *R*_1_ and *R*_4_, i.e. with an experimentally fixed negative *t*_1_, are of the so-called non-rephasing character, while *R*_2_ and *R*_3_ are of the rephasing character. Rephasing and non-rephasing signals can be measured separately. The Liouville pathways can also be grouped together according to the electronic band in which the coherences observed in 2D spectra originate. In *R*_1_ and *R*_2_, the observed coherences stem from the electronically excited state, while for *R*_3_ and *R*_4_, they come from the ground state. Thus in both rephasing and non-rephasing pairs of Liouville pathways, we observe one ground- and one excited-state coherence. It is notoriously difficult to distinguish ground- and excited-state-originated signal experimentally, unless one can find some secondary characteristics (such as signal life time due to relaxation or frequency) which distinguishes the ground- from the excited-state contributions.

Figure 2B illustrates the position, origin (excited- or ground-state manifold) and oscillating frequency of the signal generated by the different pathways in the 2D spectrum. We characterize each pathway with the superscript (*αβ*, *g*_ν_), where *α* and *β* refer to the eigenstates, and ν denotes which state, within the electronic ground-state manifold, is involved during the optical transitions. The non-rephasing pathways 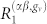 and 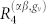 result in peaks at the position (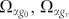) and (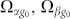), respectively, whereas the rephasing pathways 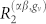 and 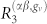 generate peaks located in similar positions, i.e. (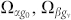). It is interesting to note the role of the index ν in the pathways. For ν = 0, there are no ground-state coherences, while excited-state coherences can appear. The frequency of oscillation of the peaks coming from 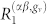 and 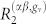 is *ω_αβ_*, while it is given by the ground-state vibrational frequency 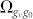 for 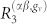 and 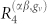. For ν > 0, the amplitudes of *R*_1_ and *R*_4_ pathways are equal, and the same holds for *R*_2_ and *R*_3_ pathways. Thus, if we would ignore the effect of the coherence life time, there would always be the same amplitude of the ground- and excited-state oscillating contributions (ν > 0), plus one contribution from the excited state (ν = 0).

Let us now discuss the (initial) amplitudes of oscillating features in 2D spectra. The initial (*t*_2_ = 0) amplitude of the third-order response function is proportional to a transition dipole moment pre-factor of the response function *R_i_* averaged over an isotropic distribution of orientations of the molecules in the sample. The dipole pre-factors *A*^(*i*)^ for each Liouville pathway *R_i_* are defined in the Supplementary Information (SI) available online. For the non-rephasing response function *R*_1_, it reads in particular 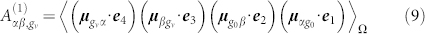
where ***e_i_*** represents the unitary orientational vector of the *i^th^* laser pulse, 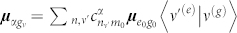 is the eigenstate transition dipole moment and 〈…〉_Ω_ stands for the averaging over an ensemble of randomly oriented complexes. The transition dipole moment on a same molecule but with different vibrational quantum number are assumed to follow the Condon approximation, i.e. 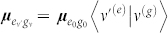. In ultrafast spectroscopy and assuming identical polarization for the four laser pulses, the disorder averaging can be accounted for by a constant orientation factor 
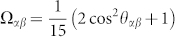
, where *θ_αβ_* is the angle between the transition dipole moments of eigenstates *α* and *β*^42^. The amplitude pre-factor then reads 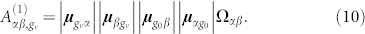
Here, 
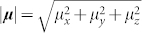
 is the length of the transition dipole moment vector.

The time-dependent response function is composed of the dipole pre-factor and the time-dependent part representing time evolution of the state of the molecular systems during the time interval between the interactions with light. The response functions *R_i_* for all pathways are presented in detail in the SI. In particular, the non-rephasing response function *R*_1_ reads as 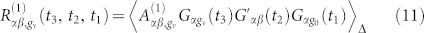
where 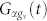 and 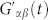 are the evolution operators of the optical coherence 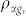 and of the excited-state coherence *ρ_αβ_*, respectively. Here, 〈…〉_Δ_ denotes averaging over the energy disorder. The details of the evolution operators are given in the SI. The contribution of the corresponding coherence to the signal in the 2D spectra is obtained after Fourier transformation of the evolution propagators during the time intervals *t*_1_ and *t*_3_: 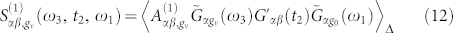
with 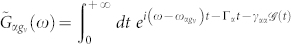 and where *ω*_1_, *ω*_3_ are the excitation and probing frequencies, respectively. The signal for the non-rephasing pathway *R*_4_, which involves the electronic ground-state coherence can be obtained in the same way. Full expressions are given in the SI.

### Application: FMO-inspired heterodimer in one particle approximation

In order to quantify the enhancement of coherence amplitude by borrowing of transition dipole moment, as illustrated by the toy model, we study a simplified molecular dimer model. In this section, we will first study the initial amplitude of the dipole pre-factor for selected coherences as a function of the molecule inter-site coupling *J* and the energy gap Δ*E*. We will include one mode of nuclear motion (*ω*_0_ = 117 cm^−1^) at each of the two sites. Then, we will investigate the dynamical behavior of the signal as a function of the dimer energy gap and the vibrational mode frequency. Possible consequences of our results for the case of FMO molecular aggregate will be addressed in the discussion.

As mentioned above, the studied dimer is inspired by the FMO lowest energy BChls 3 and 4 which are relatively strongly interacting (site coupling *J*_0_ = −53.5 cm^−1^) and form a heterodimer with an energy gap Δ*E*_0_ = 110 cm^−1^ (later referred to as the reference energy gap)^32^. The directions of the transition dipole moments were taken from the Protein Data Bank file 3ENI^43^ (we used *θ*_Bchl 3–4_ = 107.2°). A Huang-Rhys factor of *S* = 0.05 has been used to represent the generally small value experimentally measured in chlorophylls^33^. In order to assess the influence of the nuclear modes on the dimer spectrum, we studied the dependence of the spectrum on selected model parameters for a single vibrational mode. We study two cases, with frequencies *ω*_0_ = 117 cm^−1^ and 185 cm^−1^. For some parameters and spectral characteristics, we study the system properties as a function of the mode frequency in an interval with a width of roughly 200 cm^−1^.

Only one vibrational level (ν_max_ = 1) of a single mode is treated explicitly in the molecular Hamiltonian. This is motivated mainly by the small value of the Huang-Rhys factor. We have verified that we obtain similar results including a larger number of vibrational quanta. The excited-state part of the total Hamiltonian, Eq. 4, in the basis 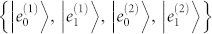 reads 
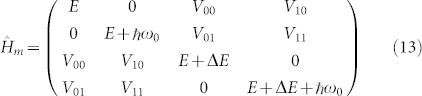
where *E* is the energy of the optical transition (*E* = 12210 cm^−1^), *V*_νν′_ = *J* (*FC*)_ν_(*FC*)_ν′_, *J* is the coupling between the two BChls and (*FC*)_ν_ = 〈*g*_0_|*e*_ν_〉 is the Franck-Condon factor characterizing the overlap of the ground-state vibrational wavefunction in the electronic ground state with that of the ν^*th*^ vibrational level in the electronic excited state. The restriction to the vibrational ground state for the (electronically) non-excited molecule follows from the one-particle approximation, detailed below.

In this work, we study the non-rephasing pathways (*R*_1_ and *R*_4_), and more specifically, their contributions to the signal on the diagonal of the 2D spectrum (*cf.* red signals in Fig. 2B). In particular, we focus on 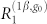 and 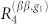 with *β* = 2 for *ω*_0_ ≤ 135 cm^−1^ and *β* = 3 otherwise (states numbered with increasing energy), because, as will be shown below, they both involve the zero- and one-phonon levels local to molecule 1, either on its electronic excited- or ground-state manifold. Comparison of the results for these two particular pathways will enable us to discuss the different characteristic of the vibronic and the ground-state vibrational coherences, thus taking into account both recent theories of the origin of the long-lived oscillations in the 2D spectra of photosynthetic systems^25^,^30^.

Unlike in Ref. 30, we concentrate on the oscillations of the diagonal peaks in the 2D spectrum, and more specifically on pathway 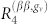 for the ground-state contributions (see Fig. 2B). Because the state |*β*〉 is initially excited on the monomer 1 and originates from the ground state of the dimer, it cannot contain an excitation of ground-state vibration on the other monomer. This allows us to stay in the one-particle approximation, where the (electronically) non-excited molecule is always considered to be in its vibrational ground state^44^. Unlike for the crosspeak, the one-particle approximation holds on the diagonal cut for all states |*β*〉 accessible from the dimer ground state. In addition, excited state absorption signal in an excitonic dimer does not contribute to the diagonal of the 2D spectrum, unless population relaxation pathways are explicitly taken into account. The population relaxation is assumed to lead to a decay of coherences only, which is described by relaxation induced dephasing rates. Correspondingly, all effects of population relaxation on relative amplitude of diagonal oscillations are contained in the stimulated emission pathway, and the excited state absorption does not have to be explicitly treated to estimate their lifetime.

### Influence of coupling and energy gap on the dipole pre-factor

**Figure 3:**
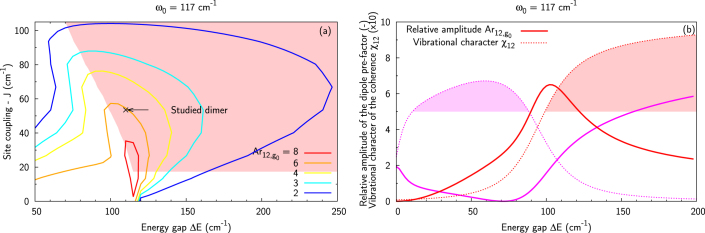
Relative amplitude of the dimer dipole moment pre-factor over that of the monomer (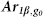) for the non-rephasing pathway *R*_1_ at initial time (a) involving coherence (1,2) as a function of the energy gap Δ*E* and coupling *J*, (b) involving coherence (1,*β*), with *β* = 2 (red) and *β* = 3 (magenta), as a function of the energy gap Δ*E* for *ω*_0_ = 117 cm^−1^. The plain thick lines represent the amplitudes, the dashed lines denote the associated measure of the vibronic character (0.1*χ*_1*β*_). In both sub-figures, the prevailingly vibrational character of the eigenstates (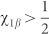) is highlighted with colored areas. A significant enhancement of the dipole moment pre-factor can be observed for coherence involving eigenstates 1 and 2, which is strongly vibrational for Δ*E* > 99 cm^−1^.

As detailed in the spectroscopy section, the initial amplitude of the signal is directly proportional to the dipole pre-factor (see Eq. 9). Here, we study the influence of the energy gap Δ*E* and site coupling *J* on the dipole pre-factor, with detailed results for pathways *R*_1_ involving the zero-phonon level of the excited state on molecule 1 (i.e. eigenstate 1, mainly localized on |*e*_1_*g*_0_〉). Figure 3 presents the relative transition dipole moment pre-factor for the dimer response function 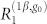 involving electronically excited eigenstate |*β*〉 = |2〉, |3〉 for *ω*_0_ = 117 cm^−1^, respective to the pre-factor corresponding to the vibrational oscillations on a monomer: 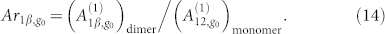
Figure 3a shows results for the coherence involving the two lowest excited eigenstates |1〉 and |2〉. Compared to the monomer, the amplitude of the dipole pre-factor in the heterodimer is clearly enhanced (up to more than 8 times) for an energy gap comparable to the vibrational energy. It should be noted that, in the range of very small couplings the excitonic model breaks down, and the electron-phonon coupling might effectively destroy the excitonic mixing. For moderate resonance coupling values and increasing its value, the region for which the amplitude is enhanced through borrowing of transition dipole moment spreads over a wider range of energy gaps corresponding to a wider mixing region. Note that the studied excited-state coherence has a prevailingly vibrational character only in the shaded area of Fig. 3a. Fig. 3b presents the amplitude of the dipole pre-factor in the coupled dimer relative to the monomeric one for coherences *ρ*_1*β*_ as a function of the energy gap Δ*E*, using *J*_0_ = −53.5 cm^−1^. We also present the local vibrational character *χ* of the coherences (*cf* Eq. 8). We can define a characteristic energy gap Δ*E_χ_* = 94 cm^−1^ for which the coherences *ρ*_12_ and *ρ*_13_ are equally delocalized, i.e. *χ*_12_ = *χ*_13_. The highest enhancement (~ 6.5 times higher than that in a monomer) is obtained for the coherence involving the two lowest excitons (1, 2). This coherence is of prevailingly vibrational character for Δ*E* > 99 cm^−1^. The coherence *ρ*_13_ exhibits only a low enhancement (< 2) in the region where it is prevailingly vibrational (10 < Δ*E* < 88 cm^−1^) and will therefore make minor contributions to long-lived coherences in the 2D spectrum.

### Amplitude of the 2D signal and life time of coherences

In this section, we study the response functions *R*_1_ and *R*_4_ involving the vibrational coherence in the electronic excited-state and in the electronic ground-state manifolds, respectively. The calculations have been performed on the FMO inspired dimer at 77 K for various vibrational frequencies, using an overdamped Brownian oscillator mode bath (see e.g. Ref. 41) with the Debye frequency Λ = 130 cm^−1^ and reorganization energy *λ* = 35 cm^−1^. Pigment energies were sampled from a Gaussian distribution with 80 cm^−1^ FWHM centered at the reference energy gap (Δ*E*_0_ = 110 cm^−1^). The energy transfer rates that contribute to the dephasing of the coherences were calculated from the same energy gap correlation functions as the line-shape functions (see SI) using the standard Redfield theory. The theory is identical with the one used in Ref. 25.

**Figure 4:**
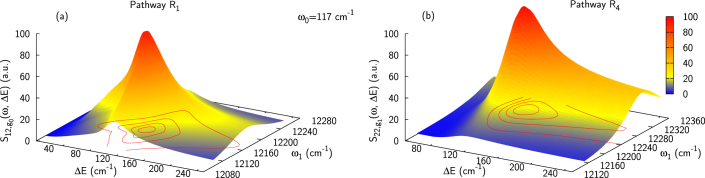
Absolute value of the oscillating signal amplitudes 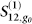 and 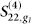 (Eqs. (15) and (16)) for pathways *R*_1_ (a) and *R*_4_ (b) as a function of the energy gap Δ*E* and excitation frequency *ω*_1_. In both pathways, the amplitude is significantly enhanced in the vicinity of the vibrational mode energy, i.e. for Δ*E* ~ *ω*_0_ (*ω*_0_ = 117 cm^−1^ here) due to the resonance occurring.

In Fig. 4, we present the energy gap dependence of the diagonal cut (*ω*_1_ = *ω*_3_) through the Fourier transform of the response functions *R*_1_ and *R*_4_ before accounting for the energy disorder: 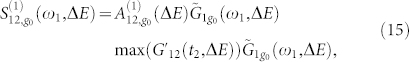

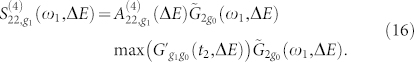
The energies of eigenstates 1 and 2 depend on both Δ*E* and *ω*_0_ (*ω*_0_ is fixed at 117 cm^−1^ here). There is a clear resonance occurring when the energy gap Δ*E* is comparable to the frequency of the nuclear mode, Δ*E* ~ *ω*_0_. This resonance demonstrates the borrowing of dipole moment from the electronic to the vibrational transition, as suggested by^25^ and illustrated by the toy model. The oscillating signal amplitude also depends on the evolution propagator 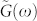 which now also depends on the mixing and therefore on Δ*E*. This dependence leads to an even larger enhancement of the oscillation amplitude (with respect to the one observed for the vibrational oscillations on a monomer) than the transition dipole moment pre-factor alone would predict.

**Figure 5:**
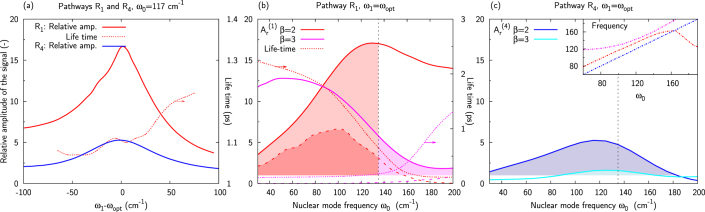
(a) Relative amplitude of the signal *A_r_* at initial time for pathways *R*_1_ and *R*_4_ (Eqs. (17) and (18)) involving the prevailingly vibrational coherence *ρ*_12_ as a function of the excitation frequency *ω*_1_ for *ω*_0_ = 117 cm^−1^. The enhancement of the vibronic coherence (pathway *R*_1_) is up to 3 times larger than that of the ground-state vibrational coherence (pathway *R*_4_). (b, c) Relative amplitude of the diagonal signal through pathways *R*_1_ (b) and *R*_4_ (c) at *ω*_1_ = *ω*_opt_ as function of the vibrational mode frequency *ω*_0_ for *β* = 2 (red, blue) and *β* = 3 (magenta, cyan). The shaded areas represent the domains in which the coherence is enhanced and of prevailingly vibrational character. The dotted dashed lines show the vibronic coherence life time, and the dashed lines represent the signal relative amplitude after 1 ps. The inset in (c) presents the frequency of the oscillating signal through the different pathways. The vertical black dashed line at *ω*_0_ = 135 cm^−1^ delimits the mode frequency at which the ordering of the eigenstates is reorganized and the vibrational character of the coherences changes. It appears that long-lived (>1 ps) vibronic coherences are created for *ω*_0_ < 120 cm^−1^ (b) whereas any mode generates long-lived ground-state vibrational coherences (c).

The complete theory of enhancement has to include averaging over the distribution of energy gaps to account for the energy disorder. Figure 5 presents the maximal amplitude of the signal from pathway *R*_1_ involving coherence *ρ*_1*β*_ relative to that of the purely vibrational coherence in a monomer (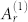, defined below), as well as that from 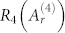, 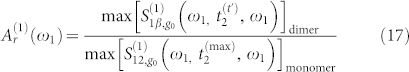

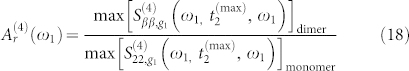
along the diagonal cut of the 2D spectrum (*ω*_3_ = *ω*_1_). The letter *β* denotes the exciton level which is composed mainly of the first vibrationally excited level located on molecule 1 (*β* = 2 for *ω*_0_ ≤ 135 cm^−1^ and 3 otherwise). 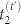 represents the time of the first maximum after a time *t*′ (*t*′ = 0 or 1 ps in the following) and 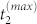 is the time at which the signal is maximum for non-decaying coherences. Figure 5a presents results for *ω*_0_ = 117 cm^−1^ (*β* = 2) as a function of the excitation frequency *ω*_1_ relatively to the frequency *ω*_opt_, which corresponds to the absorption maximum (for *ω*_0_ = 117 cm^−1^, *ω*_opt_ = 12165 and 12300 cm^−1^ for *R*_1_ and *R*_4_, respectively). For both pathways, the signal amplitude in the coupled dimer is clearly enhanced compared to that of the corresponding monomer (maximum 5 times higher for *R*_4_, and more than 16 times for *R*_1_).

Figure 5a also shows the life time of coherence *ρ*_12_ involved in *R*_1_, obtained from a fit of the calculated signal – the life time of the ground-state coherence (pathway *R*_4_) is not displayed because the purely vibrational coherences are assumed non-decaying as in Refs. 25,30. Using *ω*_0_ = 117 cm^−1^, the observed excited-state coherence appears to survive for slightly more than 1 ps.

Figure 5b shows the maximum enhancement obtained at initial time (plain lines) and after 1 ps (dashed lines) for different vibrational frequencies (on the diagonal cut at *ω*_1_ = *ω*_opt_ in the 2D spectrum) for pathway *R*_1_. Results for pathway *R*_4_ are presented in Fig. 5c. In order to account for the dependency of the eigenstate composition on the mode frequency and the resulting reordering of eigenstates at *ω*_0_ = 135 cm^−1^, results are presented for coherences *ρ*_12_ and *ρ*_13_ involved in both pathways *R*_1_ and *R*_4_. For *R*_1_, the relevant domain in which the vibronic coherence is of prevailingly vibrational character (*χ* > 0.5), is highlighted with shaded areas in Fig. 5b. For pathway *R*_4_, the ground-state vibrational coherence remains purely vibrational independently of the origin of the excited state which participates in the pathway. For both pathways, the enhancement is larger when coherence *ρ*_12_ is involved. Comparing Figs. 5b and 5c, it can be seen that, at initial time, the enhancement for pathway *R*_1_ is significantly (up to 3 times) larger than that for *R*_4_. Also, for both pathways, there is a broad resonance in the enhancement in the vicinity of the energy gap value Δ*E*_0_ = 110 cm^−1^, which is in accordance with the similarly broad resonance for the enhancement of the ground-state contribution for a crosspeak reported in Ref. 30. Because the excited-state coherences involve mixing of electronic and vibrational states, their life time depends, among others, on the dephasing time of the excited states. Consequently, long-lived (>1 ps) coherences will only be created for *ω*_0_ < 120 cm^−1^ for the pathway *R*_1_. In the case of the pathway *R*_4_, any nuclear mode will generate long-lived coherences because the purely vibrational coherences decay slowly. Therefore for higher mode frequencies, long-lived coherences should originate from pathway *R*_4_ only. We studied in detail the *R*_1_ pathway involving *ρ*_12_ using *ω*_0_ = 185 cm^−1^ (not presented here). We concluded that, although at initial time the signal is of comparable amplitude with *ω*_0_ = 117 cm^−1^, it is almost absent after 1 ps due to the decay. The inset in Fig. 5c shows the frequency of the oscillating signals. It is verified that the ground-state vibrational coherences oscillate at the frequency of the nuclear mode, whereas the frequency of the vibronic coherences does not exactly match it due to the excitonic splitting effect and the resulting dependence on the site coupling and excited-state energies.

Table I presents the contributions of the local basis excitations to the vibronic states averaged over energy disorder for *ω*_0_ = 117 cm^−1^. Eigenstate 1 consists of 85% excitation of the ν = 0 transition on site 1 and 15% on site 2. Eigenstate 2 corresponds to 64% of the ν = 1 transition on site 1. This composition explains the results presented above, and confirms that prevailingly vibrational coherences exhibit a prolonged life time.

**Figure 6:**
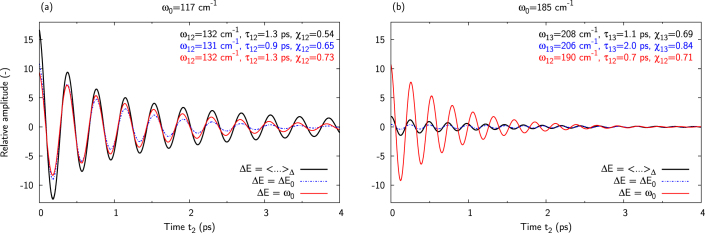
Time evolution of the signal involving coherences of prevailingly vibrational character originating in pathway *R*_1_ for two mode frequencies: *ω*_0_ = 117 cm^−1^ (a) and 185 cm^−1^ (b). The dynamics of the signal has been computed with different energy gaps, namely the energy gap averaged over the energy disorder (denoted by 〈…〉_Δ_), the resonant condition (Δ*E* = *ω*_0_) and the reference gap energy (Δ*E* = Δ*E*_0_ = 110 cm^−1^). The signal amplitude is presented relatively to that of the monomer in the region where it is maximum (*ω*_1_ = *ω*_opt_). In part (a) the mode frequency in the vicinity of the energy gap (*ω*_0_ = 117 cm^−1^) creates long-lived oscillations of significant amplitude even with energy disorder. In part (b), i. e. for a mode frequency out of resonance with the energy gap (*ω*_0_ = 185 cm^−1^), the signal benefits only sightly from the enhancement mechanism, and it remains of low amplitude.

Figs. 6a and 6b show the evolution of the signal (at *ω*_1_ = *ω*_opt_) resulting from pathway *R*_1_ and involving the prevailingly vibrational coherence for two different nuclear mode frequencies (*ω*_0_ = 117 and 185 cm^−1^), with different energy gaps, namely the reference energy gap (Δ*E*_0_ = 110 cm^−1^), the resonant condition Δ*E* = *ω*_0_, and Δ*E* sampled from the Gaussian distribution centered on the reference energy gap (denoted by 〈…〉_Δ_ in Fig. 6). The fitted frequencies and life time of the signal are indicated in the figures, along with the vibrational character *χ* of the coherence. For the mode frequency *ω*_0_ = 117 cm^−1^, we can expect long-lived oscillations with large amplitudes for any of the presented energy gaps, because the latter is either equal to or neighboring the vibrational mode frequency. Figure 6a confirms this expectation. A different behavior is observed for *ω*_0_ = 185 cm^−1^. Here, the amplitude of the vibronic coherence is significantly increased (from 0.4 to more than 10 times that in a monomer) when the energy gap corresponds to the frequency of the nuclear mode, which illustrates the resonance interaction between the two monomers. However, because the averaged energy is sampled around 110 cm^−1^, the total signal only slightly benefits from the enhancement mechanism and its amplitude is therefore smaller compared to that obtained using *ω*_0_ = 117 cm^−1^.

To sum up, the study of a model dimer has enabled us to identify the resonance between the electronic energy gap and the vibrational frequency as a crucial condition of the observation of long-lived oscillations in 2D spectra. The enhancement mechanism lies in intensity borrowing by excitonic states with strong vibrational character (the vibronic excitons) from the strongly allowed electronic states. The vibronic states thus exhibit a significant intensity despite the small Huang-Rhys factor of their vibrational component. This mechanism leads to an enhancement of the initial amplitude of both vibronic and ground-state vibrational coherences over a bandwidth of about 100 cm^−1^. However, the mixing of the electronic and vibrational character of the vibronic coherences implies that these coherences experience additional dephasing. The strong dependence of the life-time enhancement on the mode frequency means that vibronic coherences will be observable at long population times only when the frequency of the vibrational mode is close to the electronic resonance (*cf* Figs. 5b and 6). For the present model, this dephasing leads to similar amplitude of the vibronic and ground-state vibrational coherences on a picosecond timescale despite the stronger enhancement of the initial amplitude of the former. It does not therefore seem to be possible to exclude one or the other type of coherence *a priori* based on amplitude arguments. Both mechanisms will be most likely observed simultaneously in an experiment. The most useful difference between the two types of coherences is the difference in oscillation frequency (*cf* inset of Fig. 5c). The vibrational coherence oscillates with a frequency which is equal to that of the vibrational mode, while the frequency of the vibronic-exciton coherence is shifted due to the excitonic interaction.

## Discussion

The detailed investigation of the interaction between nuclear and electronic DOF in a model dimer provides more insight into the mechanism of enhancement of the amplitude and life time of the oscillations seen in 2D experiments. These results serve as a verification of the mechanism proposed by Christensson *et al.*^25^, and form a basis for the discussion of this model in relation to the one proposed by Tiwari et al.^30^. We point out that despite technical differences, such as the subtraction of the symmetric linear combination of the nuclear mode coordinates in Tiwari et al.^30^, the Hamiltonian used in these two works is the same, and so are the basics of the enhancement mechanism. The contribution of vibrational states to the optical signal is enhanced by their interaction with electronic DOF. The models differ only in the manifold in which the oscillations take place. Christensson *et al.*^25^ suggested the excited state, while Tiwari et al.^30^ studied the ground-state contribution. As we have illustrated above, the amplitude of both ground-state vibrational and vibronic coherences is significantly enhanced in a system of coupled molecules as compared to the isolated monomers. The recent 2D experiments on BChls in solution did not find any significant vibrational coherences^45^. These results are in line with what one would expect based on the Huang-Rhys factors of the low frequency vibrational modes determined by previous experiments. However, in aggregates, an enhancement by factor of five at 1 ps was found for both excited- and ground-state contributions, strong enough to elevate the signal above the noise level.

Another mechanism of mode-driven coherences has also been recently proposed, where electronic coherences are assumed to emerge from resonant coupling with robust vibrational coherences^29^. The constant transfer from the long-lasting vibrational coherences is assumed to maintain long-lived oscillations on the otherwise fast dephasing electronic coherences. However, one should point out that this study uses Huang-Rhys factors about one order of magnitude higher than in the present paper, and that vibrational transitions are not expected to be as weak as with the parameters used here.

To distinguish ground- and excited-state contributions to non-linear optical signals is notoriously difficult, and to identify unique signatures of electronic, vibronic-exciton and vibrational coherence is less than trivial^29^,^46^,^47^,^48^,^49^. To distinguish between electronic and vibrational coherences, a recent research suggested the use of broadbrand pump/broadband probe spectroscopy, showing that oscillations as function of the waiting time originate from electronic oscillations in the singly excited manifold^50^. Another promising proposal has been the use of specific polarization sequences^51^, which could further be used to perform quantum process tomography^52^. It has been shown that a specific combination of polarizations in the four wave mixing sequence can single out coherences involving transitions with non-zero angle in the molecular frame^53^,^54^. When the vibrational modes are treated as members of the bath (i.e. standard exciton picture), vibrational transitions on a single pigment are always parallel, and the orientational average for pathways representing vibrational coherences will be zero. Therefore such polarization sequences are termed “coherence specific” sequences and have been assumed to only report on electronic coherences^51^,^54^. However, when the vibrational modes are treated explicitly, the mixing between vibrational and electronic DOF leads to a non-zero angle between the vibronic states, even those largely located on the same pigment. The vibronic coherences (pathway *R*_1_) discussed in Ref. 25 will thus not be eliminated by the coherence specific polarization sequence. The same argument applies to the ground-state vibrational coherences. When modes are treated explicitly, there will be a nonzero angle between 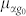 and 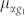, and pathways of type *R*_4_ will also contribute to the signal^30^. We can thus conclude that the “coherence specific” sequence is not able to unambiguously distinguish between electronic, vibronic and ground-state vibrational coherence.

To be able to determine the origin of the oscillations observed in the experiments, additional arguments, such as the oscillation frequencies or dephasing times, are needed. The ground-state vibrational coherences will oscillate with a frequency equal to that of the relevant vibrational mode, while for the vibronic coherences, the oscillation frequency will depend on the vibrational frequency as well as on the electronic coupling and site energies. The vibronic coherences also experience additional dephasing due to the mixing of electronic and vibrational DOF, while the ground-state vibrational coherences only experience vibrational dephasing. These quantitative differences could in principle be used to distinguish the two models, if the vibrational frequencies, Huang-Rhys factors and dephasing times of the vibrational modes were known.

The low frequency vibrational spectrum of BChl-a in solutions or in different protein environments have been studied by a number of techniques. Studies of isolated BChl-a in frozen solution have demonstrated that the strongest vibrational mode is found around 180-190 cm^−1^ 55,56,57,58. These experiments have identified a mode around 160 cm^−1^, but its Huang-Rhys factor was found to be significantly weaker than the one of the modes around 180–190 cm^−1^ 55,56. Although the results of the experiments are rather consistent, it should be pointed out that the low-frequency spectral range is sensitive to the local conformation of the macrocycle and the coordination of the central Mg atom. This effect was illustrated by Rätsep *et al.*^55^ by measuring the fluorescence line narrowing (FLN) spectra of BChl-a in different solvents. These experiments demonstrate that results obtained on the isolated pigment might not be a good indicator of the frequencies and Huang-Rhys factors observed in the protein environment.

A widely studied case of “isolated” BChl-a in a protein environment is the accessory BChl in the bacterial reaction center. Resonance Raman (RR) spectra of the accessory BChl (B-band) have been reported by several groups^59^,^60^,^61^. The room temperature spectrum identified modes at 84, 117, 180 and 210 cm^−1^, where the latter two were considerably stronger^60^. Measurements at 95 K revealed an additional mode at 160 cm^−1^ which was ~10 times weaker than the mode at 180 cm^−1^ 60. Similar results were obtained at even lower temperature, showing that modes around 180 and 220 cm^−1^ dominate the spectrum^61^.

There is only a limited number of studies on the vibrational spectrum of the pigments in the FMO complex. A hole burning (HB) study found two modes at 120 and 160 cm^−1^ with a comparable strength, but were not able to observe any modes with higher frequencies due to the narrow absorption band in FMO^62^. FLN measurement, on the other hand, revealed a spectrum which was similar to the RR spectrum of the B-band in the reaction center^33^,^63^. The two FLN measurements on the different species are similar, showing a mode at 117 cm^−1^ and several modes between 167–202 cm^−1^. The strongest modes in both cases were found around 190 cm^−1^.

Taking all of the data on the low frequency vibrational spectrum of BChl-a, we can conclude that none of the reported spectra show the presence of a single strong mode in the frequency range corresponding to that observed in the 2D experiments (160 cm^−1^). Rather, the spectra reveal a large number of vibrational modes with comparable Huang-Rhys factors in the range from 80–240 cm^−1^ corresponding to typical energy splittings in FMO^32^. All these modes will thus, to a certain extent, experience enhancement due to the interaction between electronic and vibrational DOF. For the ground-state wave-packets, the frequency of the oscillation will match that of the vibrational mode. Based on the discussion above, we would predict that the mode at 117 cm^−1^ experiences the strongest enhancement. A rather broad amplitude enhancement curve furthermore implies that multiple oscillation frequencies should be observed. For the vibronic coherences, the observed frequency depends on the resonance coupling as well as on the vibrational frequencies, the number of pigments and probably also on the number of vibrational modes included in the Hamiltonian. For the dimer used here, we predict an oscillating frequency of 132 cm^−1^ and for the full model, the value of 140 cm^−1^ was found^25^. Increasing the resonance coupling would blue-shift the oscillation frequency further and increase the amplitude of the vibronic coherence. However, due to the increased electronic character, these coherences would also experience a stronger dephasing and a correspondingly shorter life time. Because the amplitude enhancement, oscillation frequencies and dephasing dynamics are all closely related to the electronic structure, a simulation including all pigments is required to determine whether it is possible to find a set of couplings which results in a strong enhancement, correct oscillation frequency and a long life time. Such a simulation could also determine whether vibronic or ground-state vibrational coherences dominate in the case of FMO. Furthermore, it would be possible to elucidate why a single oscillation dominates in the 2D spectrum, although multiple vibrational modes are expected to experience significant amplitude enhancement according to the reduced dimer models.

In conclusions, we have demonstrated on a model dimer how the interaction between electronic and nuclear degrees of freedom leads to enhanced amplitudes of both the excited-state vibronic-exciton coherences and the ground-state vibrational coherences in 2D spectra. The results obtained highlight the properties of the enhancement mechanism. The vibronic-exciton model used here also provides, without contradiction with molecular dynamics simulations, a plausible explanation for correlated fluctuations in different spectral regions, often postulated in purely excitonic model to reproduce long-lived coherences.

We find that the initial amplitude of the vibronic-exciton and vibrational coherences are enhanced by up to 15 and 5 times, respectively, compared to the vibrational coherences in the isolated monomer, with a bandwidth of about 100 cm^−1^ for both mechanisms. This enhancement requires both sufficient electronic coupling and a resonance between the vibrational level on one pigment and the electronic transition on the other. Enhancement is found to be even more pronounced when the inhomogeneity of the sites is included. For the dimer model used in this work, although the vibronic-exciton coherences exhibit a larger initial amplitude compared to the ground-state vibrational coherences, we find that both types of coherences have a similar magnitude at long (picosecond) population times. Whether the vibronic coherences dominate in 2D spectra or not depends on the excited-state dephasing and population dynamics as well as the influence of inhomogeneous broadening. To decide which type of coherence dominates in FMO complex would require simulations with all seven pigments and a full spectral density including a larger spectrum of vibrational modes.

## Author Contributions

T.M. and N.C. created the model and detailed the theoretical methods together with A.C. who performed the calculations and interpretation. N.C. discussed the extension of results to FMO. A.C., N.C., H.F.K. and T.M. participated in writing and reviewing the paper.

## Supplementary Material

Supplementary Information Vibronic and Ground-state vibrational coherences in 2D spectra of photosynthetic complexes (supplementary information)

## Figures and Tables

**Table 1:** Composition of the eigenstates and eigenenergies averaged over the energy disorder for *ω*_0_ = 117 cm^−1^. Significant contributions are highlighted with bold font

		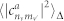		*α* = 1	*α* = 2	*α* = 3	*α* = 4
n = e	m = g	ν = 0	ν*′* = 0	**0.85**	0.08	0.07	0.00
		ν = 1		0.00	**0.64**	**0.36**	0.00
n = g	m = e	ν = 0	ν*′* = 0	0.15	**0.29**	**0.57**	0.00
			ν*′* = 1	0.00	0.00	0.00	**0.99**
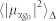				1.64	0.16	0.16	0.00
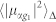				0.05	0.48	0.47	0.82
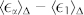		(cm^−1^)		0.	128.	163.	294.
