# Supplementary material for: Enhancement of Vibronic and Ground-State Vibrational Coherences in 2D Spectra of Photosynthetic Complexes
Source: Sci Rep. 2013 Jun 19;3:2029. doi: 10.1038/srep02029 (PMC3693153; doi:10.1038/srep02029)
Supplement: Supplementary Information — Vibronic and Ground-state vibrational coherences in 2D spectra of photosynthetic complexes (supplementary information) [file srep02029-s1.pdf]

Enhancement of Vibronic and Ground-State Vibrational Coherences in 2D Spectra of Photosynthetic Complexes (Supplementary Information)

Aurélia Chenu[1], Niklas Christensson[2], Harald F. Kauffmann[2], and Tomáš Mančal[1]
[1]Faculty of Mathematics and Physics, Charles University,
Ke Karlovu 5, 121 16 Prague 2, Czech Republic and
[2]Faculty of Physics, University of Vienna, Strudlhofgasse 4, 1090 Vienna, Austria

Third-Order Response Function

In this supplementary information, we detail the dipole pre-factors and time-dependent response functions for each Liouville pathways $R_1 - R_4$.

The transition dipole moment pre-factors read

$$A^{(1)}_{\alpha\beta,g_\nu} = \langle(\boldsymbol{\mu}_{g_\nu\alpha}\cdot\boldsymbol{e}_4)(\boldsymbol{\mu}_{\beta g_\nu}\cdot\boldsymbol{e}_3)(\boldsymbol{\mu}_{g_0\beta}\cdot\boldsymbol{e}_2)(\boldsymbol{\mu}_{\alpha g_0}\cdot\boldsymbol{e}_1)\rangle_\Omega \quad (1)$$

$$A^{(2)}_{\alpha\beta,g_\nu} = \langle(\boldsymbol{\mu}_{g_\nu\beta}\cdot\boldsymbol{e}_4)(\boldsymbol{\mu}_{\alpha g_\nu}\cdot\boldsymbol{e}_3)(\boldsymbol{\mu}_{\beta g_0}\cdot\boldsymbol{e}_2)(\boldsymbol{\mu}_{g_0\alpha}\cdot\boldsymbol{e}_1)\rangle_\Omega \quad (2)$$

$$A^{(3)}_{\alpha\beta,g_\nu} = \langle(\boldsymbol{\mu}_{g_\nu\beta}\cdot\boldsymbol{e}_4)(\boldsymbol{\mu}_{\beta g_0}\cdot\boldsymbol{e}_3)(\boldsymbol{\mu}_{\alpha g_\nu}\cdot\boldsymbol{e}_2)(\boldsymbol{\mu}_{g_0\alpha}\cdot\boldsymbol{e}_1)\rangle_\Omega \quad (3)$$

$$A^{(4)}_{\alpha\beta,g_\nu} = \langle(\boldsymbol{\mu}_{g_0\beta}\cdot\boldsymbol{e}_4)(\boldsymbol{\mu}_{\beta g_\nu}\cdot\boldsymbol{e}_3)(\boldsymbol{\mu}_{g_\nu\alpha}\cdot\boldsymbol{e}_2)(\boldsymbol{\mu}_{\alpha g_0}\cdot\boldsymbol{e}_1)\rangle_\Omega, \quad (4)$$

where $\langle\ldots\rangle_\Omega$ denotes the averaging over a random orientation of the pigments.

The time-dependent response functions read:

$$R^{(1)}_{\alpha\beta,g_\nu}(t_3,t_2,t_1) = \left\langle A^{(1)}_{\alpha\beta,g_\nu}\, G_{\alpha g_\nu}(t_3)\, G'_{\alpha\beta}(t_2)\, G_{\alpha g_0}(t_1)\right\rangle_\Delta \quad (5)$$

$$R^{(2)}_{\alpha\beta,g_\nu}(t_3,t_2,t_1) = \left\langle A^{(2)}_{\alpha\beta,g_\nu}\, G_{\beta g_\nu}(t_3)\, G'_{\beta\alpha}(t_2)\, G_{g_0\alpha}(t_1)\right\rangle_\Delta \quad (6)$$

$$R^{(3)}_{\alpha\beta,g_\nu}(t_3,t_2,t_1) = \left\langle A^{(3)}_{\alpha\beta,g_\nu}\, G_{\beta g_\nu}(t_3)\, G'_{g_0 g_\nu}(t_2)\, G_{g_0\alpha}(t_1)\right\rangle_\Delta \quad (7)$$

$$R^{(4)}_{\alpha\beta,g_\nu}(t_3,t_2,t_1) = \left\langle A^{(4)}_{\alpha\beta,g_\nu}\, G_{\beta g_0}(t_3)\, G'_{g_\nu g_0}(t_2)\, G_{\alpha g_0}(t_1)\right\rangle_\Delta, \quad (8)$$

with $\langle\ldots\rangle_\Delta$ representing the averaging over a random distribution of pigment energies. The evolution propagators of the optical and excited-state coherences, $G(t)$ and $G'(t)$, respectively, read

$$G_{\alpha g_\nu}(t) = e^{-i\omega_{\alpha g_\nu}t}e^{-\Gamma_\alpha t}e^{-\gamma_{\alpha\alpha}\mathscr{G}(t)}, \quad (9)$$

and

$$G'_{\alpha\beta}(t) = e^{-i\omega_{\alpha\beta}\,t}e^{-(\Gamma_\alpha+\Gamma_\beta)\,t}e^{-(\gamma_{\alpha\alpha}+\gamma_{\beta\beta}-2\gamma_{\alpha\beta})\,\mathscr{G}(t)}, \quad (10)$$

where $\omega_{\alpha\beta} = \frac{\epsilon_\alpha-\epsilon_\beta}{\hbar}$ is the energy difference between the two eigenstates α and β, Γ_α is the eigenstate relaxation rate computed from the Redfield tensor and $\mathscr{G}_{\alpha\beta}(t) = \gamma_{\alpha\beta}\,\mathscr{G}(t)$ denotes the line-shape function. The γ coefficients can be expressed, in one particle approximation, via the expansion coefficients $c^\alpha_{n_\nu m_0}$ as

$$\gamma_{\alpha\beta} = \sum_{n,\nu}\left(c^\alpha_{n_\nu m_0}\right)^2\left(c^\beta_{n_\nu m_0}\right)^2 + \sum_{n,\nu>\nu'}\left[\left(c^\alpha_{n_\nu m_0}\right)^2\left(c^\beta_{n_{\nu'} m_0}\right)^2 + \left(c^\alpha_{n_{\nu'} m_0}\right)^2\left(c^\beta_{n_\nu m_0}\right)^2\right]. \quad (11)$$

Using the Debye spectral density, $\tilde{C}''(\omega) = \pi\omega^2 J(\omega) = 2\lambda\frac{\omega\Lambda}{\omega^2+\Lambda^2}$, where λ is the bath reorganization energy and Λ the Debye frequency, the line-shape function reads, in high-temperature approximation, [1]

$$\mathscr{G}(t) = \left(\frac{2\lambda k_B T}{\hbar\Lambda} - i\frac{\lambda}{\Lambda}\right)\left(e^{-\Lambda t} + \Lambda t - 1\right), \quad (12)$$

with k_B the Boltzmann constant and T the temperature.

The contributions of a particular coherence to the signal in a 2D spectrum is obtained after Fourier transformation of the evolution propagators during the time intervals t_1 and t_3:

$$S^{(1)}_{\alpha\beta,g_\nu}(\omega_3,t_2,\omega_1) = \left\langle A^{(1)}_{\alpha\beta,g_\nu}\,\tilde{G}_{\alpha g_\nu}(\omega_3)\,G'_{\alpha\beta}(t_2)\,\tilde{G}_{\alpha g_0}(\omega_1)\right\rangle_\Delta \tag{13}$$

$$S^{(2)}_{\alpha\beta,g_\nu}(\omega_3,t_2,\omega_1) = \left\langle A^{(2)}_{\alpha\beta,g_\nu}\,\tilde{G}_{\beta g_\nu}(\omega_3)\,G'_{\beta\alpha}(t_2)\,\tilde{G}_{g_0\alpha}(\omega_1)\right\rangle_\Delta \tag{14}$$

$$S^{(3)}_{\alpha\beta,g_\nu}(\omega_3,t_2,\omega_1) = \left\langle A^{(3)}_{\alpha\beta,g_\nu}\,\tilde{G}_{\beta g_\nu}(\omega_3)\,G'_{g_0 g_\nu}(t_2)\,\tilde{G}_{g_0\alpha}(\omega_1)\right\rangle_\Delta \tag{15}$$

$$S^{(4)}_{\alpha\beta,g_\nu}(\omega_3,t_2,\omega_1) = \left\langle A^{(4)}_{\alpha\beta,g_\nu}\,\tilde{G}_{\beta g_0}(\omega_3)\,G'_{g_\nu g_0}(t_2)\,\tilde{G}_{\alpha g_0}(\omega_1)\right\rangle_\Delta \tag{16}$$

where $\tilde{G}_{\alpha g_\nu}(\omega) = \int_0^{+\infty} dt\, e^{i(\omega-\omega_{\alpha g_\nu})t-\Gamma_\alpha t-\gamma_{\alpha\alpha}\mathscr{G}(t)}$ and ω_1, ω_3 are the excitation and probing frequencies, respectively. After analytical integration, we obtain

$$\tilde{G}_{\alpha g_\nu}(\omega) = \frac{e^{A_0}}{\Lambda A_0^{A_\alpha(\Delta\omega)}}\left[\Gamma\left(A_\alpha\left(\Delta\omega\right)\right) - \Gamma\left(A_\alpha\left(\Delta\omega\right),\, A_0\right)\right], \tag{17}$$

where $\Delta\omega = \omega_{\alpha g_\nu} - \omega$ and $A_0 = \frac{2\lambda k_B T}{\Lambda^2} - i\frac{\lambda}{\Lambda}$ are constants, and $A_\alpha(\omega) = \frac{\Gamma_\alpha - i\omega}{\Lambda} - A_0$. $\Gamma(z)$ is the Euler gamma function with complex argument which satisfies $\Gamma(z) = \int_0^{+\infty} t^{z-1}e^{-t}dt$ and $\Gamma(a,z) = \int_z^{+\infty} t^{a-1}e^{-t}dt$ is the incomplete gamma function.

---

[1] S. Mukamel, *Principles of nonlinear spectroscopy* (Oxford University Press, Oxford, 1995).
